# Reduction of Involuntary Admissions in Patients With Severe Psychotic Disorders Treated in the ACCESS Integrated Care Model Including Therapeutic Assertive Community Treatment

**DOI:** 10.3389/fpsyt.2019.00736

**Published:** 2019-10-24

**Authors:** Daniel Schöttle, Friederike Ruppelt, Benno G. Schimmelmann, Anne Karow, Alexandra Bussopulos, Jürgen Gallinat, Klaus Wiedemann, Daniel Luedecke, Anja Christine Rohenkohl, Christian G. Huber, Thomas Bock, Martin Lambert

**Affiliations:** ^1^Psychosis Centre, Department of Psychiatry and Psychotherapy, University Centre of Psychosocial Medicine, Medical Center Hamburg-Eppendorf, Hamburg, Germany; ^2^University Hospital of Child and Adolescent Psychiatry, University of Bern, Bern, Switzerland; ^3^University Hospital of Child and Adolescent Psychiatry, University Centre of Psychosocial Medicine, Medical Center Hamburg-Eppendorf, Hamburg, Germany; ^4^Department of Adult Psychiatry, Universitäre Psychiatrische Kliniken Basel (UPK), University of Basel, Basel, Switzerland; ^5^Department of Psychiatry and Psychotherapy, University Centre of Psychosocial Medicine, Medical Center Hamburg-Eppendorf, Hamburg, Germany

**Keywords:** psychosis, involuntary admissions, coercive, multiple episodes, follow-up

## Abstract

**Objective:** The ACCESS treatment model offers assertive community treatment (ACT) embedded in an integrated care program to patients with severe psychotic disorders. Compared to standard care, it proved to be more effective in terms of service disengagement and other outcomes in patients with psychotic disorders over 12, 24, and 48 months. Many patients with severe mental disorders experience involuntary admissions which can be potentially traumatic. In this study, we assessed the effect of ACT on reducing involuntary admissions over an observation period of 4 years.

**Method:** One hundred seventy-one patients treated in ACCESS were included in this study. The primary outcome was rate of involuntary admissions during 48 months. Secondary outcomes were differences between those with and without involuntary admissions in the 2 years prior to ACCESS regarding change of psychopathology, severity of illness, psychosocial functioning, quality of life, satisfaction with care, medication non-adherence, and service-disengagement.

**Results:** Of 171 patients, 58 patients (33.9%) were involuntarily admitted to hospital in the past 2 years before entry. During the 4 years of treatment, 16 patients (9.4%) were involuntarily admitted to hospital which was a significantly lower rate compared to the 2 years before inclusion in ACCESS (p < .001). Comparing the two groups, larger improvements in severity of illness (p = .004) and functional status (p = .043) were detected in the group with no history of involuntary admissions. At 4-year follow-up, of the remaining patients, 69.2% (n = 81) were full adherent (p < .001), compared to 18.9% (n = 31) at baseline with no differences between the two groups over the study period (p = .25). Over 4 years, only 13 patients (13.2%) were service-disengaged due to non-practical reasons.

**Conclusions:** In this long-term study, we were able to demonstrate a reduction in involuntary admissions in four treatment years compared to the 2 years prior to admission to the ACCESS model in patients with severe and mostly multiphase schizophrenia spectrum disorders and affective disorders with psychotic features. This may help prevent patients from suffering from a potentially traumatic experience during treatment in the psychiatric system.

**Clinical Trial Registration:**www.ClinicalTrials.gov, identifier NCT01888627.

## Introduction

With the progressive deinstitutionalization of psychiatric care, outpatient care has changed significantly in recent decades. The treatment of patients with the most severe forms of mental disorders, such as psychotic disorders (1, 2), is still demanding. Patients often remain very susceptible to future recurrences after a first episode and experience persistent or even increasing difficulties in symptoms and functioning, even when they are not acutely ill (2, 3). Schizophrenia spectrum and bipolar disorders (especially with comorbid substance use disorders) are associated with one of the highest risks of involuntary hospitalization for patients (4, 5). Compulsorily admitted patients often lack insight into their disorders and the need for treatment (6).

In addition, patients with schizophrenia spectrum or bipolar disorders often experience other forms of coercive treatment, such as seclusion, mechanical restraint, or coercive medication, or more subtle forms, such as informal coercion (7–9). Therapeutic staff have to deal with the difficult ethical and clinical task of patient care on the one hand, and with respect for patients’ autonomy in trying to maintain a good therapeutic relationship between these conflicting requirements on the other. Therapeutic self-understanding does not include the use of institutional violence, sometimes necessary to prevent patients from harming themselves or others (10). Even when coercive measures are used to regain patients’ autonomy and enable individuals to recover from a severe psychotic episode—for example, coercive treatment of patients is often experienced negatively and may be traumatic (11–13). A negative attitude and acceptance of future psychiatric care, unfavorable treatment courses with high relapse rates, and subsequent involuntary admissions as well as rejection of inpatient and outpatient treatment may possibly develop as negative consequences of such a loss of existential autonomy and coercion (10, 13–19). In addition, the experience and/or use of violence in a psychiatric treatment environment can be potentially negative and even traumatic for therapeutic personnel (20). Apart from those who perceive their coercive treatment as negative, there are a considerable number of patients who consider it to be justified retrospectively. Patients’ perceptions of coercive experiences depend, among other things, on when patients are asked about a coercive event, the therapeutic relationship, how coercive measures are communicated to patients, and as how fair and effective they are experienced (12, 21–26). Studies with results on long-term outcomes on other variables such as treatment discontinuation, symptomatology, and functional status are sparse and have shown heterogeneous findings.

In recent years, various outpatient care models have been developed for patients with severe mental illnesses (SMI), which are adapted to their complex treatment needs and generally show positive effects. Most of them comprise multiprofessional teams and individualized, flexible, and domestic treatments such as assertive community treatment (ACT), flexible ACT (FACT), and intensive case management (ICM) (27–35). There are also other approaches, such as the Crisis Resolution Team, which offer temporary treatment (36).

Most study care models are diagnosis-specific and do not provide continuous and unlimited treatment for patients with severe mental disorders (32).

Because patients with SMI have high rates of withdrawal, non-adherence, involuntary admission, and often chronic disease progression, specific, timely, and permanent treatment may be required for patients with psychotic disorders beyond early detection of new episodes and pure crisis management (33, 37). In addition, treatment must overcome structural barriers and fragmentation of treatment systems to ensure therapeutic continuity. The therapeutic alliance depends, among other things, on continuous confidence-building and long-term treatment. Since the therapeutic relationship is one of the most effective factors for successful treatment, this must be ensured (38). In 2006, our group designed and evaluated a diagnosis-specific integrated treatment model with ACT (the ACCESS model) specializing in psychotic disorders (rather than critically ill patients in general), with a focus on maintaining a continuous therapeutic relationship, low-threshold psychotherapy, and family involvement and embedding the ACT team in an integrated care program that allows for need-adapted, time-unlimited treatment (27, 39). Under real-life conditions, the effectiveness of the program was continuously evaluated, and the results were published in various studies (27, 29, 39–49). The evidence of a reduction in involuntary admissions or the use of coercive measures in intensive care models is difficult to compare and ambiguous due to methodological differences in treatment systems used. It is known that unfavorable therapeutic conditions such as high barriers to access to psychiatric care, the availability of home treatment, or crisis intervention teams have an impact on the rate of involuntary admissions (50–52). In addition, involuntary admissions often take place in hours outside regular outpatient services (50–52). Early detection of an emerging episode to prevent a new crisis or rapid worsening of symptoms, combined with early involvement of family members or friends in the home environment, may be key elements of assertive outreach treatment. Although this could theoretically lead to prevention of involuntary admission due to early and rapid treatment, assertive outreach teams also attract critically ill patients who not only have a higher chance of involuntarily being admitted to hospitals but who are better able to recognize the need for treatment that, conversely, can increase or have no effect on the rate of (involuntary) admission (36, 53–56).

Although intensive treatment models can have positive effects on, amongst others, symptomatology, relapses, and hospital stay, there are few studies that assess their direct effects on involuntary admissions with heterogeneous outcomes, partly due to methodological differences, differences between national legislations, and model adherence in the implementation of assertive outreach structures (29, 49, 55, 57–61).

In our present study, we report the frequency of involuntary admission of 171 patients with severe psychotic disorders during 4 years of treatment compared to 2 years of treatment before admission to ACCESS. In addition, we performed outcome comparisons between a group that had involuntary admission and a group that had not been involuntarily admitted to hospital in the last 2 years prior to ACCESS in terms of outcome variables such as course of psychopathology, severity of disease, functional status, quality of life, and satisfaction with treatment. We assume that the rate of involuntary admissions would decrease compared to the 2 years prior to admission over an extended period of 4 years of treatment and that both groups would show similar improvements throughout the study period.

## Methods

### Context, Sample, and Inclusion and Exclusion Criteria

The Psychosis Center of the University Hospital Hamburg-Eppendorf is responsible for the treatment of adult patients with severe schizophrenia spectrum disorders (SSD) or bipolar disorder (BD) in an urban catchment area of 300,000 inhabitants.

The ACCESS model is described in detail elsewhere (27) (29). The main features of the integrated care concept, including details on ACT, inclusion and exclusion criteria, and assessments, are presented in **Table 1** and **Table 2**. From May 2007 to March 2012, 171 patients with SSD and BD and severe mental illness were included within the ACCESS model. Of these, 171 patients who were followed-up for 4 years were analyzed. All treated patients (N = 171) participated in the clinical routine assessments. The study was conducted in accordance with the latest version of the Helsinki Declaration, and written informed consent of the participants was obtained. All patients treated in the ACCESS model agreed that their data could be used in the ACCESS-II study whenever they were sufficiently stable, and the ability to consent was determined by a counseling psychiatrist. The ethics committee of the Hamburg Medical Association approved the observational study (registration number: PV4059). The study was registered at ClinicalTrials.gov (NCT0188868627).

**Table 1:** Characteristics of the ACCESS treatment and inclusion/exclusion criteria.

Characteristics	Content
**Integrated care model**	
**Catchment area with population size**	• Catchment area of the Department of Psychiatry and Psychotherapy of the University Medical Center, 300,000 habitants
**Health care facilities within the IC model**	• Specialized psychosis inpatient unit with attached day-clinic; acute inpatient unit (closed ward), specialized psychosis outpatient center, ACT team, specialized day-clinic for first-episode psychosis patients in the age range of 15–29, working support outpatient center, 20 private psychiatrists
**ACT team fidelity**	
**Maximum full-time equivalent caseload**	• 15–25
**Staff fidelity and skills**	• Consultant psychiatrists, psychiatrists, psychologists, nurses, social worker
**Staff skills**	• Diagnosis-specific training in pharmacotherapy, cognitive behavioral (CBT), dynamic, and/or family psychotherapy, pharmacotherapy
**Work style**	• Shared caseload, patients are discussed in daily team meetings, weekly internal and external supervisions, regularly patient-centered network meetings
**Availability**	• Extended hours (8 a.m. to 6 p.m. Monday to Friday) and 24-hour crisis telephone and 24-hour emergency service within the Department
**Contact with clients**	• High frequency face-to-face contacts, assertive engagement, shared-decision making, “no drop-out” policy
**Main interventions**	• Case management; home treatment; individual, group, and family psychotherapies; psychoeducation; pharmacotherapy; social work
**Inclusion and exclusion criteria**	
**Inclusion criteria:**	• Diagnosis of a schizophrenia-spectrum disorder (i.e., schizophrenia, schizophreniform disorder, schizoaffective disorder, delusional disorder, or psychotic disorder not otherwise specified) or bipolar disorder with psychotic features, all assessed with the Structured Clinical Interview for DSM-IV Axis I Disorders (SCID-I) (62) Aged ≥18 years Present hospitalization because of an acute illness state as assessed by a psychiatrist Presence of a certain severity of illness as assessed with the Brief Psychiatric Rating Scale, 24-item version (BPRS),(63) with (1) BPRS total score ≥40 points and (2) fulfillment of 1 of the following eight criteria: ≥6 points on item 10 (hallucinations); ≥6 points on item 11 (unusual thought content); ≥6 points on item 15 (conceptual disorganization); ≥ 10 total points on items 3 and 4 (depressive-suicidal syndrome); ≥6 points on item 4 (suicidality); ≥15 total points on items 8, 9, and 21 (manic syndrome); ≥15 total points on items 6, 12, and 20 (disruptive behavior syndrome); or ≥15 total points on items 13, 16, and 17 (negative syndrome).
**Exclusion criteria:**	• Psychotic disorders due to a medical condition were excluded.

**Table 2:** Assessments and measures.

Assessments and measures	Details
**Fidelity of the ACT team**	Fidelity of the team to the assertive community treatment model was assessed yearly with the Dartmouth Assertive Community Treatment Scale (64). The DACTS has 28 criteria and 3 subscales [(1) human resources: structure and composition, (2) organizational boundaries, (3) nature of services]. The maximal score on the DACTS is 5, representing a perfect implementation of all ACT principles. At initiation of ACCESS, the total score was 4.5 and varied yearly between 4.2 and 4.6 points, indicating that fidelity of the treatment model was good.
**Fidelity of ratings**	Trained raters independent of the treatment team to avoid bias. All raters received extensive training, particularly for SCID-I interviews, BPRS, CGI-S, and GAF.
**Assessment time points**	Baseline, week 6, and months 3, 6, 12, 18, 24, 30, 36, 40, and 48
**Diagnoses**	Diagnoses of the psychotic disorder and comorbid axis I disorder(s) were assessed with the SCID-I (62).
**Service disengagement**	Service disengagement for non-practical reasons was considered to be present if a patient repeatedly refused further treatment despite the need and several attempts of reengagement (phone calls to patient and potentially home visits by the assertive community treatment team) (33).
**Service use data**	Treatment contacts consisted of face-to-face meetings as well as emails/letters, telephone calls, and contact with institutions or family members. Furthermore, hospital days (inpatient and day-clinic treatments) were recorded for each year of treatment. All service use data are presented for patients being actively treated in each year (i.e., excluding service-disengaged patients).
**Baseline assessments**	• Sociodemographic, functional, and pretreatment characteristics using the German version of the Early Psychosis File Questionnaire (65) • Employment/occupation using the Modified Vocational Status Index (MVSI) (66) and the Modified Location Code Index (MLCI) (66). “Employed/occupied” comprised paid or unpaid full- or part-time employment, being an active student in university, a full- or part-time volunteer; “independent living” comprised living alone, with a partner, or with peers. The MVSI and MLCI are scales rated from 1 to 7, with lower scores indicating a better vocational status and a better ability to live independently. • Duration of untreated psychosis with the Duration of Untreated Psychosis Scale (67–69) • Prevalence of previous inpatient treatment, lifetime involuntary admission, and admission within the 2 years before ACCESS were assessed by interviewing patients, relatives, and health service staff previously responsible for the patient. Data were validated by cross checking the hospital database. Involuntary admissions were due to danger to self or others. • Medication adherence was assessed using the criteria of Kane et al. (70). Therapists rated their patients as being fully adherent in the last 4 weeks if taking ≥ 80% of their prescribed medications, partially adherent when taking 20–80%, and nonadherent when taking ≤ 20% of the prescribed medications.
**Baseline and follow-up assessments**	• Psychopathology using the BPRS at baseline and every 6 months • Severity of illness using the Clinical Global Impressions—Severity of Illness scale (CGI-S) (71) • Level of functioning using the Global Assessment of Functioning (GAF) Scale(72) • Quality of life using the 18-item Quality of Life Enjoyment and Satisfaction Questionnaire (QLES-Q-18) (73) • Patients’ satisfaction with their care using the Client Satisfaction Questionnaire (CSQ-8) (74) • Medication adherence (see previous paragraph above) (70)

To evaluate involuntary admissions before and after inclusion in the ACCESS program, we used a pre-post-mirror comparison design. We decided to evaluate a longer observation period of 4 years in order to better detect differences between the time before and after admission and between the two groups. Involuntary admissions were assessed 2 years before admission and during the 4-year observation period. Involuntary treatment included compulsory admission based on (1) the “Hamburger PsychischKrankenGesetz” (HambPsych KG; §12 and §9) with patients who meet the criteria of acute risk to themselves or others from a mental disorder initiated by a physician or psychiatric hospital ordered by law, or (2) the Bürgerliches Gesetzbuch (BGB) with compulsory admission initiated by a parent or guardian under §1906. While both actions require a very acute symptomatology, compulsory admissions of the second type cannot be performed if there is solely a danger for others. Both actions, which lead to involuntary admissions to hospitals, require the opinion of a psychiatrist and must be approved by a judge.

### Statistical Analysis

Descriptive baseline differences between diagnostic groups were assessed *via* independent-samples t-tests for continuous dependent variables. Categorical variables were assessed with chi-square tests. To compare baseline with the 48-month follow-up for the binary outcomes (e.g., involuntary admissions), we used McNemar’s test. We evaluated the changes from baseline (admission to ACCESS treatment) *via* mixed-model repeated measures, considering the follow-up times as repeated measures, the patients as the random effect, the group (with *vs.* without involuntary admissions 2 years before baseline) and time as fixed effects, and the baseline values of the dependent variable as covariates. Outcomes were changes from baseline for BPRS total score, CGI-S score, GAF, Q-LES-Q-18, and CSQ-P. We examined the interaction between time and group. If the interaction was not significant, the interaction term was eliminated from the model. We used the baseline values of the dependent variables (BPRS total score, CGI-Severity score, GAF, Q-LES-Q-18, CSQ-8 P) as covariates to minimize variance (75). The main effects (F), significance levels (p), and estimated marginal means (EMM) and 95%-confidence intervals (CI) are reported. The level of significance was set at p < .05, two-sided. Statistical analyses were performed using SPSS version 20.0 (IBM Corp, 2011).

## Results

### Baseline Characteristics

One hundred and seventy-one patients with SSD or BD (42.7% male; mean age = 42.3 years; SD 13.4) were treated in the ACCESS model and participated in the ACCESS-II study. Baseline details are displayed in **Table 3**.

**Table 3:** Baseline variables.

Demographic details	All patients(N = 171)	No history of involuntary treatment (n = 113)	2 years before ACCESS history of involuntary treatment (n = 58)	p-value
Age, mean (SD)	42.31 (13.36)	40.49 (12.70)	45.86 (14.01)	.012*
Sex, n (%), male	73 (42.7)	45 (39.8)	28 (48.3)	.329
Partnership, n (%), single	115 (67.3)	78 (69.0)	37 (63.8)	.304
Education years, mean (SD)	11.08 (1.80)	11.21 (1.83)	10.81 (1.74)	.168
Completed professional education, n (%)	110 (64.3)	76 (67.3)	34 (58.6)	.321
Employment/occupation, n (%)	30 (17.5)	23 (20.4)	7 (12.1)	.207
Living independently, n (%)	154 (90.1)	100 (88.5)	54 (93.1)	.426
**Illness details**				
First episode psychosis, n (%)	26 (15.2)	21 (18.6)	5 (8.6)	.115
Affective psychosis, n (%)	52 (30.4)	31 (27.4)	21 (36.2)	.292
Comorbid psychiatric disorder at entry, n (%)	156 (91.2)	102 (90.3)	54 (93.1)	.776
Substance use disorder (SUD) lifetime, n (%)	117 (68.4)	72 (63.7)	45 (77.6)	.082
Other comorbid disorder lifetime, n (%)	132 (77.2)	88 (77.9)	44 (75.9)	.848
Comorbid somatic disorders at entry, n (%)	138 (80.7)	91 (80.5)	47 (81.0)	1.00
Family history of psychiatric disorder^a^				
Any psychiatric disorder, n (%)	89 (52.0)	60 (53.6)	29 (52.7)	1.00
Psychotic disorder, n (%)	43 (25.1)	30 (26.8)	13 (23.6)	.710
Insight into illness before IC, n (%)	106 (62.0)	72 (64.3)	34 (58.6)	.507
Suicide attempts in the past, n (%)	68 (39.8)	44 (38.9)	24 (41.4)	.869
Suicidal thoughts at entry, n (%)	67 (39.2)	43 (38.1)	24 (41.4)	.741
Forensic history, n (%)	13 (7.6)	9 (8.0)	4 (7.1)	1.00
Traumatic adversities				
Any traumatic adversity in the past, n (%)	118 (69.0)	80 (70.8)	38 (67.9)	.724
Traumatic adversities before age 18, n (%)	96 (56.1)	62 (54.9)	34 (58.6)	.745
Duration of untreated illness				
DUP, median in weeks (quartiles)	21.86 (8.57; 56.64)	24.79 (8.43; 55.36)	21.57 (8.65; 104.29)	.719
DUP, week mean (SD)	57.97 (82.43)	56.34 (80.66)	61.18 (86.44)	
DUI, week mean (SD)	212.94 (211.84)	231.61 (261.53)	176.27 (199.09)	.109
DUI, median in weeks (quartiles)	152.14 (52.21; 280.50)	162.57 (53.07; 329.21))	104.43 (52.14; 230.36)	
Full adherence with last medication, n (%)	31 (18.1)	26 (24.3)	5 (8.6)	.020*
**Baseline scores of assessment scales**				
BPRS total score, mean (SD)	79.71 (19.37)	77.48 (18.50)	84.07 (20.44)	.035*
CGI-S-score, mean (SD)	5.79 (0.90)	5.76 (0.86)	5.84 (0.95)	.561
GAF-score, mean (SD)	36.16 (11.24)	36.57 (10.88)	35.36 (11.96)	.509
Q-LES-Q-18-score, mean (SD)	2.26 (0.66)	2.27 (0.68)	2.25 (0.62)	.881
CSQ-8 P-score, mean (SD)	2.78 (0.59)	2.78 (0.59)	2.80 (0.60)	.818

DUP, duration untreated psychosis; DUI, duration untreated illness; BPRS, Brief Psychiatric Rating Scale; CGI-S, Clinical Global Impression scale—Severity score; GAF, Global Assessment of Functioning scale; CSQ-8 P, Client Satisfaction Questionnaire-8 (patient version); Q-LES-Q-18, Quality of Life Enjoyment and Satisfaction Questionnaire. ^a^First and second-degree relatives. *p < .05.

Of all 171 patients, 23 (13%) did not have any psychiatric inpatient treatment before inclusion in the ACCESS model. Furthermore, as 141 (85%) of the patients already had multiple episodes of their illness, we assume that most of them had contact to the treatment system and had been treated with different forms of psychopharmacological and psychotherapeutic interventions. However, we could not assess this question in detail, as detailed data on prior outpatient treatment were not available.

Patients with both schizophrenia-spectrum disorders (n = 147) and BD (n = 24) were severely ill (high CGI-S and BPRS scores and low GAF scores). Quality of life and satisfaction with care before entry into the ACCESS treatment model were low; 58 patients (34%) had involuntary admissions to inpatient treatment in the past 2 years before inclusion in ACCESS, and only 18.1% (n = 31) were adherent to their most recent medication. Patients with a recent history of involuntary admissions were significantly older (45.9 *vs.* 40.5 years; p = 0.012), less adherent (8.6 *vs.* 24.3%; p = 0.02) with the last medication, and had higher scores on the BPRS Scale (84.1 *vs.* 77.5; p = 0.035) than the patient group without involuntary admissions.

### Rates of Involuntary Admissions During 4 Years of Treatment

Of those being involuntarily treated in the past 2 years before entry, 47 patients had a SSD (32.0% of the whole SSD-group) and 11 patients were diagnosed with a BD with psychotic symptoms (45.8% of the whole BD-group) (p = .184). During the 4 years of treatment, 16 patients (9.4%) were involuntarily admitted to hospital which was a significantly lower rate (58 patients; 34%) compared to the 2 years before inclusion in ACCESS (p < .001). Of those being involuntarily admitted during 4 years of treatment, 14 patients had a diagnosis of a SSD (9.5% of the whole SSD-group) and 2 patients had BD with psychotic symptoms (8.3% of the whole BD-group).

### Clinical Course of Patients With and Without Involuntary Admissions in the 2 Years Prior to ACCESS

All follow-up assessments during the 4 years indicated significantly improved psychopathology, illness severity, global functioning, and quality of life in patients (**Table 4**). Comparing the two groups, larger improvements in severity of illness (p = .004) and functional status (p = .043) were detected in the group with no history of involuntary admissions 2 years before ACCESS, compared to the group without involuntary admissions, between baseline and year 4. No significant differences were found on the course of psychopathology and quality of life. Regarding satisfaction with treatment, the CSQ-8 scores indicated a significantly better than baseline satisfaction with care, with a mean rating of “good” at 12- and 24- and 48-month follow-ups with no differences between the groups.

**Table 4:** Course of illness over 4 years.

Measure	Baseline		24-month follow-up		48-month follow-up		MMRM		
	No	Yes	No	Yes	No	Yes	Time effect, F	Group effect, F	Time x Group, F
BPRS total score, M (SD)	77.5 (18.5)	84.1 (20.4)	48.5 (9.9)	52.6 (14.1)	46.2 (8.9)	51.0 (13.6)			
EMM, SE			−28.8 (1.0)		−31.2 (1.1)		12.1***	0.7	ns (1.5)
CGI-Severity score, M (SD)	5.8 (0.9)	5.8 (1.0)	3.9 (0.9)	4.1 (1.1)	3.6 (1.0)	3.8 (1.1)			
EMM, SE			−3.2 (0.2)	−3.0 (0.3)	−4.1 (0.2)	−4.8 (0.3)	17.1***	0.2	2.7**
GAF, mean (SD)	36.6 (10.9)	35.4 (12.0)	60.7 (11.0)	57.1 (13.0)	65.0 (12.2)	61.2 (13.6)			
EMM, SE			23.8 (1.7)	19.9 (1.6)	28.3 (1.3)	24.5 (1.9)	12.6***	3.8	1.9*
Q-LES-Q-18, M (SD)	2.3 (0.7)	2.3 (0.6)	3.3 (0.6)	3.3 (0.5)	3.4 (0.5)	3.3 (0.6)			
EMM, SE			1.0 (0.1)		1.1 (0.1)		4.2***	0.1	ns (1.7)
CSQ-8 P, M (SD)	2.8 (0.6)	2.8 (0.6)	3.2 (0.5)	3.3 (0.5)	3.2 (0.4)	3.3 (0.5)			
EMM, SE			0.4 (0.0)		0.4 (0.1)		1.5	0.6	ns (0.9)

BPRS, Brief Psychiatric Rating Scale; CGI-S, Global Clinical Impression scale—Severity score; GAF, Global Assessment of Functioning scale; Q-LES-Q-18, Quality of Life Enjoyment and Satisfaction Questionnaire; CSQ-8 P, Client Satisfaction Questionnaire-8 (patient version); M, mean; SD, standard deviation; EMM, estimated marginal mean; SE, standard error. *p < .05; **p < .01; ***p < .001.

At 48-month follow-up, of the remaining patients, 69.2% (n = 81) were full adherent (McNemar’s test, p < .001), compared to 18.9% (n = 31) at baseline with no differences between the two groups over the study period (p = 0.25).

Furthermore, in the whole group, significantly more patients were employed/occupied after 48 months (n = 35; McNemar’s test, p = 0.036), while rates of living independently remained stable (n = 88, p = .332). There were no significant differences between the two groups regarding both variables.

### Service-Disengagement

Over the 48-month treatment period, 13 patients (13.2%) were service-disengaged after a median of 79.1 weeks (quartiles 36.9–150.6) due to non-practical reasons (refused treatment contact, disengaged from study despite several attempts to engage them). Of these 13 patients, 8 (61.5%) had an involuntary admission in the 2 years before ACCESS and 3 (23.1%) during the ACCESS treatment. Furthermore, 41 patients (24.0%) dropped out of the study due to practical reasons [moved out of catchment area: 15 patients (36.6%); moved to sheltered housing: 13 patients (31.7%); transition to other service: 10 patients (24.4%); change of health insurance company: 1 patient (2.4%); change of diagnosis: 2 patients (4.9%) after a median duration of treatment of 91.4 weeks (quartiles 40.9-130.1)]. Of these 41 patients, 13 (31.7%) had an involuntary admission in the 2 years before ACCESS and 10 (24.4%) during the ACCESS treatment.

## Discussion

The ACCESS model provides treatment as a temporally unlimited care model and is delivered to a sample of critically ill patients, especially with recurring SSD and BD with complex treatment needs.

In this study, we focused (1) on the rates of involuntary admissions during long-term treatment and (2) on whether those with or without involuntary admissions prior to ACCESS differ in other outcome parameters such as symptomatic progression, functional status, quality of life, and satisfaction with treatment. In addition, we analyzed differences on employment and living standards.

We were able to show that the rate of involuntary admissions decreased significantly during ACCESS treatment over 4 years. While in the 2 years prior to ACCESS one in three patients experienced involuntary admission, the rates were reduced to 9.4% over 4 years of treatment. Most patients involuntarily admitted to hospital were diagnosed with SSD, which is consistent with other studies showing that patients with SSD belong to a high-risk group for involuntary admissions. Compared to the group of patients with SSD, the rates of those with BD have decreased significantly compared to the 2 years prior to ACCESS treatment, so it appears that these patients benefit particularly from the need-adapted and fast-response treatment system that offers high-frequency home treatment, including psychotherapy and early family involvement. Our results are consistent with other studies showing a reduction of coercive measures during intensive outpatient treatment (29, 49) (58, 61), but the results of other studies are heterogeneous (56, 60). These are due to methodological differences, e.g., different treatment approaches/systems, different national legislations, patient characteristics, follow-up time, model fidelity, and therefore difficult to compare. Nevertheless, the CRT in the study by Johnson et al. did not lead to a reduction in involuntary detentions, and no difference in coercive measures was found in the OPUS study, in which ACT was offered, and compared with usual treatment (55, 59). The availability of low-threshold and high-frequency outpatient treatment and crisis resolution teams led to a reduction in involuntary admissions in the study by Juckel et al. (52, 62). On the other hand, there are studies showing an increase in the frequency of involuntary hospital admissions (56, 60), probably due to the group of patients (1) in which mainly seriously ill patients at high risk of involuntary admission are treated and (2) in which those who would normally not reached by the psychiatric treatment network can be identified and then treated, often with a first compulsory hospital treatment. In addition, there is a group of patients who would not have entered the psychiatric treatment system under “treatment as usual” circumstances and who are difficult to treat even in intensive care approaches, but who are nevertheless contacted by assertive outreach teams and then voluntarily admitted to hospital.

Although causal attributions are not possible due to the absence of a control group, we assume that some factors contribute to the observed significant decreases in compulsory hospital admissions for patients with complex treatment needs, high co-morbidity rates, and high chances of treatment discontinuation and non-adherence (49).

It can only be assumed which of the factors are related to the reductions of admissions. We believe that treatment should be offered openly and need-adapted with a small enough case load per case manager to allow for multiple outpatient contacts per week. The treatment team should be committed to psychotherapy and family involvement and should be recovery oriented. The most important points are the therapeutic alliance and the ability to intervene early, both factors being related to the high-intensity and ongoing treatment.

Although we lack specific empirical evidence from our data, continuous treatment seems especially important with a high number of treatment contacts, leading to a well-established treatment alliance between patients and their therapists, the low-threshold availability of the assertive outreach team, with rapid detection and response to emerging crises, is among the key elements of ACCESS that in our view contribute to reducing involuntary admissions in our patients. In addition, the early and intensive involvement of family members and other key individuals, as well as the recovery-oriented psychotherapeutic approach, may further have contributed to promoting treatment engagement. Other factors mentioned in the study by Burns et al., which do not explicitly contribute to a reduction in compulsory admission but in number of inpatients days, can also be found in our treatment approach: regular home visits, a high proportion of home contacts, smaller patient-to-therapist ratio, responsibility for health and social affairs, and multidisciplinary teams (76).

Confirming the results of our previous study, the psychopathology of the patients, the severity of the disease, the functioning, and the quality of life improved overall during the 4-year treatment period (29, 49). Although difficult to compare with each other because of differences in sample composition and the offered care model itself, other trials have shown that intensive treatment can improve and stabilize patients with severe mental illness—as long as it is actively and continuously offered (75, 77). We cannot deduce causality from our non-randomized single-group design, but we believe it is worth providing affordable and flexible but highly specific long-term care for patients with first and multiple episode psychosis (78). The group with involuntary admissions prior to ACCESS had fewer improvements in severity and functional status than the other patients. The only fundamental difference we found was that these patients were older, had higher baseline values on the BPRS scale, and had lower adherence rates to their previous medication. Non- or partial adherence with psychopharmacological treatment is one of the major risk factors for relapses (79, 80), which significantly reduces patients’ psychosocial and occupational functioning and negatively affects their quality of life (79, 81, 82). Psychopharmacological treatment, as an integral part of an integrated framework for social and psychological care, can help to overcome these impairments and is highly effective (83). Non-adherence with treatment is, however, particularly frequently observed in patients with schizophrenia (80, 84, 85) with a significantly increased risk of relapse (86). Even small gaps in medication intake can have a negative impact on the outcome, since discontinuation of medication for only 1 to 10 days in a period of 1 year (partial adherence) was associated with a significantly increased risk of hospitalization with a quota ratio of 1.98 (87). Partial adherence, such as intermittent medication intake, also leads to a 3-fold higher relapse risk in stable patients (88). Adherence rates increased significantly during treatment, and we found no persistent differences in medication use between the two groups.

Quality of life was not significantly influenced by previous involuntary admissions, a finding also found in other studies (89, 90). In the study by Ohlenschlaeger et al., the patients in integrated treatment, who are not directly comparable to our study due to the focus on patients of the first episode, showed a better quality of life compared to other treatment models (91).

Satisfaction with the treatment was also “good” in all patients without group differences. Treatment satisfaction is influenced by many factors but seems to be more related to the subjectively perceived degree of coercion during admission and treatment than to the objective (documented) extent of coercive measures (92). We did not measure levels of perceived coercion, but it is interesting in the context of intensive assertive outreach treatment that, among other factors, viewing the hospital as ineffective and other treatments as more appropriate and involving patients in the decision-making and treating them with respect may reduce perceived coercion (93). As our treatment model involves patients intensively in decision-making, this could have influenced ratings of satisfaction with treatment.

The disengagement rate of services over 48 months remained very low over the 4 years at 13.2%, which was slightly higher than in the previous 4-year study (8.7% disengagement rate). Insofar, in the years since the beginning of ACCESS, we had a constant afflux of patients. Therefore, the team increased from one multiprofessional team consisting of 4 full-time team members in 2007 treating 64 patients to 10.7 full-time team members in the year 2019, who work in 3 multiprofessional teams treating 228 patients. We were creating new small teams to achieve that every team member knows each patient and to make sure that personal treatment continuation is guaranteed.

### Strengths and Limitations

Due to the observational, non-randomized study design, more severely ill patients with higher rates of comorbidities were included, who probably would not have provided consent to participation in a (randomized) controlled trial. During such a long follow-up period, it was possible to assess the long-term effects of continuous treatment beyond the initial course of illness. The biggest limitation, of course, is the lack of a control group in the ACCESS-II study. Therefore, a direct causal effect of the treatment program on the results of the key outcome parameters cannot be drawn. Instead, other factors may also be responsible for the positive results found in patients treated in the ACCESS model over 4 years. Therefore, the descriptive results must be interpreted with cautions. We decided, after a prospectively controlled study confirmed the superiority of the ACCESS model over standard treatment over a period of 1 year, that only an observational and uncontrolled long-term study can be considered ethical. Another unavoidable limitation was the non-blind assessment of patients. Although we have used external advisors to ensure the quality of evaluation, we probably could not completely avoid a social desirability bias and thus positive evaluations of psychopathology. One major outcome—the rate of involuntary admissions—was not influenced by social desirability or nonblind assessments. However, patients who dropped out due to non-practical reasons could have impaired the beneficial results since it is not known whether they would have been involuntarily admitted during the observation period. This cannot be ruled out, but it seems unreasonable because they dropped out after having been in treatment for almost 2 years. Since the sample size of the involuntary treatment group is rather small, analyses of differences between the two groups may be underestimated. In addition, we did not include homeless people, so the sample is not fully representative and is limited by the exclusion of homeless people. These were treated elsewhere by definition of the catchment area. In addition, we cannot exclude the possibility that other important confounding factors were not assessed, including the specific impact of different psychopharmacological treatments. In our treatment model, patients are actively engaged to participate in treatment decisions, and dose-reductions are facilitated in close consultation with the therapist. Therefore, it is not likely that the results are due to general increases in doses of outpatient medication.

## Conclusion

In this long-term study, we were able to demonstrate a reduction in involuntary admissions in four treatment years compared to the 2 years prior to admission to the ACCESS model in patients with severe and mostly multi-episode SSD and affective disorders with psychotic features. This may help prevent patients from suffering from a potentially traumatic experience during treatment in the psychiatric system. The ACCESS model, which was offered in a timely and unlimited manner, provided results related to several clinically important outcome parameters, with low disengagement and significantly improved medication adherence rates. We hypothesize some factors to explain these positive outcomes. Psychosis-specific ACT, embedded in an integrated care system that offers a wide range of treatment options for psychotic disorders and comorbidities flexibly and rapidly, with a focus on recovery-oriented psychotherapy and family involvement, could have contributed to strengthening the therapeutic alliance that, together with the above-mentioned treatment system, could serve as a protective factor. Treatment should be offered on a need-adapted basis with a low caseload to allow a high frequency of contacts. While the results are promising, to draw causal conclusions, stronger evidence including a long-term RCT would be required. Nevertheless, our study adds important knowledge that there is an association of intensive and ongoing home treatment and a significant reduction of involuntary admissions during long-term treatment of patients with severe mental illness.

## Data Availability Statement

The full dataset of the analysis is not publicly available due to the rules of data protection of our hospital.

## Ethics Statement

The ethics committee of the Hamburg Medical Association approved the observational study (registration number: PV4059). The study was registered under ClinicalTrials.gov (NCT0188868627).

## Author Contributions

Conceptualization: DS, FR, TB, AK, ML; data curation: FR; data analyses: FR; investigation: DS, FR, AB, JG, KW, TB, AK, ML; methodology: BS, FR, project administration: JG, AK, ML; supervision: ML; writing original draft: DS, FR, AK, ML; writing, review and editing: DS, ML, FR, AB, JG, KW, AR, CH, TB, AK; contribution: all authors contributed to manuscript revision, read and approved the submitted version.

## Conflict of Interest

DS has been a consultant and/or advisor to or has received honoraria from Astra Zeneca, Otsuka Pharma GmbH, Lundbeck GmbH, Janssen Cilag and Shire.BS has been a consultant and/or advisor to or has received honoraria from Shire, Takeda, Novartis and Medice.TB has received honoraria from Astra Zeneca.ML has held lectures for AstraZeneca, Bristol-Myers Squibb, Lilly Deutschland GmbH, Janssen Cilag GmbH, Lundbeck GmbH, Otsuka Pharma GmbH, Roche Deutschland Holding GmbH, Sanovi Aventis; Grants: AstraZeneca, Bristol-Myers Squibb, Lilly Deutschland GmbH, Janssen Cilag GmbH, Lundbeck, Sanovi Aventis; Consultancy: AstraZeneca, Lilly Deutschland GmbH, Janssen Cilag GmbH, Roche Deutschland Holding GmbH, Trommsdorff GmbH & Co. KG. AK has been consultant and/or advisor to or has received honoraria from: AstraZeneca, Bristol-Myers Squibb, Lilly Deutschland GmbH, Janssen Cilag GmbH, Lundbeck GmbH, Otsuka Pharma GmbH, Roche Deutschland Holding GmbH.JG has received research funding from the German Federal Ministry of Education and Research, German Science Foundation, and speaker fees from Lundbeck, Janssen-Cilag, Lilly and Otsuka.CH serves in the cariprazine advisory board for Recordati AG and has received travel grants from Recordati AG, Servier (Suisse) S.A., and Janssen-Cilag AG, Switzerland.KW has been a consultant and received honoraria for lectures from Janssen GmbH, Lundbeck GmbH and Otsuka Pharma GmbH. DL has served as speaker for Janssen Cilag.

The remaining authors declare that the research was conducted in the absence of any commercial or financial relationships that could be construed as a potential conflict of interest.
